# Circulating Microvesicles Enriched in miR–126–5p and miR–223–3p: Potential Biomarkers in Acute Coronary Syndrome

**DOI:** 10.3390/biomedicines13020510

**Published:** 2025-02-18

**Authors:** José Rubicel Hernández-López, Mirthala Flores-García, Esbeidy García-Flores, Benny Giovanni Cazarín-Santos, Marco Antonio Peña-Duque, Fausto Sánchez-Muñoz, Martha Alicia Ballinas-Verdugo, Hilda Delgadillo-Rodríguez, Marco Antonio Martínez-Ríos, Eduardo Angles-Cano, Aurora de la Peña-Díaz

**Affiliations:** 1 Pharmacology Department, Faculty of Medicine, National Autonomous University of Mexico, Circuito Escolar, Ciudad Universitaria, Coyoacán, Mexico City 04510, Mexico; rubicel1981@hotmail.com (J.R.H.-L.); benny.cazarin@facmed.unam.mx (B.G.C.-S.); 2 Molecular Biology Department, National Institute of Cardiology Ignacio Chávez, Juan Badiano 1, Tlalpan, Mexico City 14080, Mexico; mirthala.flores@cardiologia.org.mx (M.F.-G.); esbeidy.garcia@quimica.unam.mx (E.G.-F.); 3 Biochemistry Department, Faculty of Chemistry, National Autonomous University of Mexico, Circuito Escolar, Coyoacán, Mexico City 04510, Mexico; 4 Cardiology Service, Medica Sur. Puente de Piedra 150, Toriello Guerra, Tlalpan, Mexico City 14050, Mexico; marcopduque@gmail.com; 5 Physiology Department, National Institute of Cardiology Ignacio Chávez, Juan Badiano 1, Tlalpan, Mexico City 14080, Mexico; fausto22@yahoo.com; 6 Immunology Department, National Institute of Cardiology Ignacio Chávez, Juan Badiano 1, Tlalpan, Mexico City 14080, Mexico; ballinasv75@gmail.com; 7 Department of Hospitalization, National Institute of Cardiology Ignacio Chávez, Juan Badiano 1, Tlalpan, Mexico City 14080, Mexico; hilda_car@yahoo.com; 8 Independent Researcher, Tlalpan, Mexico City 14050, Mexico; mtzrios@gmail.com; 9 INSERM UMR_S-1140 & UMR_S-1144, Innovation Diagnostique et Thérapeutique en Pathologies Cérébrovasculaires et Thrombotiques, Faculté de Pharmacie de Paris, Université Paris Cité, 75006 Paris, France

**Keywords:** miRNAs, microvesicles, acute coronary syndrome, TIMI, biomarkers

## Abstract

**Background.** The molecular mechanisms underlying acute coronary syndrome (ACS) have been extensively investigated, with a particular focus on the role of circulating microvesicles (MVs) as carriers of regulatory elements that influence hemodynamic changes and coronary flow. Endothelial and platelet dysfunction during ACS alters MV composition, impacting clinical outcomes. This study explores the levels of miR–126–5p and miR–223–3p in circulating MVs and their association with the Thrombolysis in Myocardial Infarction (TIMI) coronary flow classification scale, proposing their potential as biomarkers. **Methods.** Bioinformatic tools identified miRNAs linked to ACS. Plasma MVs were isolated from ACS patients and healthy controls through high-speed centrifugation. miRNA levels were quantified using quantitative reverse transcription polymerase chain reaction (qRT-PCR) and compared across TIMI 0 and TIMI 3 groups. Diagnostic efficacy was assessed via receiver operating characteristic (ROC) curve analysis. **Results.** The bioinformatic analysis identified miR–126 and miR–223 present in ACS. miR–126–5p and miR–223–3p were significantly reduced in MVs from TIMI 0 patients compared to TIMI 3. ROC analysis showed high diagnostic accuracy for miR–126–5p (AUC = 0.918; 95% CI: 0.818–1.00; *p* = 0.001) and miR–223–3p (AUC = 1.00; 95% CI: 1.00–1.00; *p* < 0.001). **Conclusions.** Reduced levels of miR–126–5p and miR–223–3p in circulating MVs are strongly associated with impaired coronary flow, positioning these miRNAs as potential biomarkers for ACS risk stratification and therapeutic targeting.

## 1. Introduction

Acute coronary syndrome (ACS) encompasses a spectrum of clinical conditions caused by insufficient coronary blood flow, including acute myocardial infarction (AMI) and unstable angina (UA) [1]. ACS is a leading cause of morbidity and mortality worldwide, with cardiovascular diseases responsible for over 17.9 million deaths annually, according to the World Health Organization (2023) [2]. This high prevalence underscores the urgent need for improved diagnostic and prognostic tools to better understand ACS pathophysiology and enhance patient care.

Coronary thrombosis is a critical event in ACS and results from a complex interplay between endothelial cells and platelets. Under normal physiological conditions, the endothelium maintains vascular homeostasis by releasing anti-thrombotic factors such as nitric oxide (NO) and prostacyclin (PGI2). However, when endothelial injury occurs, often due to atherosclerotic plaque rupture, subendothelial matrix proteins such as von Willebrand factor and collagen become exposed, initiating platelet adhesion and activation [3]. Platelets rapidly respond by secreting pro-thrombotic molecules, including thromboxane A2 (TXA2) and adenosine diphosphate (ADP), further amplifying platelet aggregation via the glycoprotein IIb/IIIa (GPIIb/IIIa) receptor. This cascade results in thrombus formation, which can occlude the coronary artery, leading to myocardial ischemia and infarction. The extent of vascular occlusion and the ability of endogenous or therapeutic thrombolytic mechanisms to restore blood flow define the patient’s prognosis, which is often assessed using the Thrombolysis in Myocardial Infarction (TIMI) scale [3].

In this context, the TIMI coronary flow grade represents a key clinical tool for assessing ACS severity into four grades: TIMI 0 (no perfusion), TIMI 1 (minimal perfusion), TIMI 2 (reduced-speed perfusion), and TIMI 3 (normal perfusion) [4]. Stratification using the TIMI scale has been linked to clinical outcomes such as reinfarction [5], mortality [6,7], ventricular aneurysm formation [8], and arrhythmias [9]. Achieving TIMI 3 flow is a critical benchmark for therapeutic success [10,11]. Despite advances in reperfusion therapies and pharmacological treatments, ACS remains a clinical challenge due to its complex and multifactorial nature. The lack of reliable biomarkers to identify molecular and hemodynamic changes in ACS limits clinicians’ ability to accurately predict outcomes [12].

In the last decade, several prospective studies have examined the relationship of baseline circulating microRNA (miRNA) levels with the risk of myocardial infarction or cardiovascular events (Zampetaki et al. (2012) [13], Yuan et al. (2014) [14], Jansen et al. (2014) [15], Bye et al. (2016) [16], Gigante et al. (2020) [17], Keller et al. (2017) [18], Velle-Forbord et al. (2019) [19]) (see Supplementary Material). Discrepancies in results are attributed to differences in populations (age, sex, risk factors and diagnosis), study methods (RT-qPCR, microchips, arrays), sample type (plasma, serum or microvesicles (MVs)), normalization and study design with various statistical analyses. Notably, few studies correlated results with the TIMI scale, both acute and stable coronary events were included, and miRNAs were identified in plasma, serum or MVs.

Circulating MVs have emerged as promising biomarkers and mediators in cardiovascular disease. MVs are extracellular vesicles released by platelets [20], endothelial cells [21], and leukocytes [22] in response to stress or activation. They carry bioactive molecules, including proteins, lipids, and miRNAs [23]. miRNAs, small non-coding RNAs (21–25 nucleotides), regulate gene expression post-transcriptionally by targeting messenger RNA for degradation or translational repression, influencing up to 60% of human genes [24]. In ACS, miRNAs such as miR–26, miR–126, miR–133, miR–144, miR–208, miR–223, and miR–483 modulate platelet activity, oxidative stress, cardiac remodeling, and inflammation [25,26]. MVs have been implicated in endothelial dysfunction, platelet activation, and disrupted coronary blood flow [27,28,29]. These vesicles facilitate platelet–platelet and platelet–endothelial communication, regulating thrombotic processes [30,31,32].

Alterations in MV cargo, including miRNAs, during ACS reflect underlying pathophysiological processes. This study focuses on miR–126–5p and miR–223–3p levels in circulating MVs and examines their potential as biomarkers for ACS, particularly in relation to the TIMI flow scale.

## 2. Materials and Methods

### 2.1. Study Population

This cross-sectional convenience study, conducted in 2018, included 32 patients diagnosed with ACS. The patients were recruited from the Hemodynamics Department of the National Institute of Cardiology (INC) Ignacio Chávez, Mexico City. Inclusion criteria were age ranging from 40 to 75 years, a diagnosis of UA, and ST-elevation myocardial infarction (STEMI), or non-ST-elevation myocardial infarction (NSTEMI) confirmed via angiography according to the American College of Cardiology criteria [33]. The study included 9 patients with UA, 10 with STEMI, and 13 with NSTEMI. These patients were stratified based on TIMI flow grade by an expert cardiologist from the Hemodynamics Department. This stratification accounted for the impact of biological and mechanistic factors associated with full reperfusion as a comparative group (TIMI 3, 17 patients) and lack of reperfusion (TIMI 0, 15 patients). A control group of 10 healthy individuals aged 40 to 75 years recruited from the INC blood bank was included during the same time frame as the patient cohort. To minimize bias, our study compared TIMI 0 versus TIMI 3 patients rather than comparing against a group of healthy individuals. This approach ensured that both groups received the same medication regimen and hospital treatment, reducing confounding variables. In this sense, our sample size was determined based on feasibility constraints, including the availability of participants and the specific inclusion criteria applied in our study. Exclusion criteria included autoimmune, hepatic, renal, or oncological diseases. All participants provided informed consent, and the study was approved by the Research and Ethics Committees of INC with registration number FIMICOR 18-1043.

### 2.2. Microvesicles Extraction

Blood samples were collected in sodium citrate (0.109 M, relation 1:9) tubes and centrifuged at 1500× *g* for 15 min to obtain plasma. Plasma was further centrifuged at 13,000× *g* for 2 min and stored at −80 °C. MVs were isolated through high-speed centrifugation (20,000× *g* for 90 min, 4 °C) and resuspended in 100 µL of phosphate buffer, were added to 400 µL of QIAzol Lysis Reagent and stored in aliquots at –20 °C before RNA extraction.

### 2.3. RNA Extraction and Quantitative Reverse Transcription Polymerase Chain Reaction (RT-qPCR)

RNA was extracted from MVs using the Direct–zol^TM^ RNA MiniPrep Kit (Zymo Research, Irvine, CA, USA), according to the manufacturer’s instructions, and was eluted in 25 µL of RNase–free water. RNA samples were stored at –80 °C until processing. For miRNA complementary DNA (cDNA), we employed the TaqMan© MicroRNA Reverse Transcription Kit (Applied Biosystems, Foster City, CA, USA), following the manufacturer’s instructions. Retrotranscription was developed according to the manufacturer’s instructions. The results were analyzed and samples with amplification after 35 cycles were discarded. miRNA levels were quantified using TaqMan© miRNA Assays (Applied Biosystems, Foster City, CA, USA) for miR–126–5p, miR–223–3p, and miR–39–3p as control were employed in a Roche LightCycler^®^ 480–II instrument (Roche Applied Sciences, Beijing, China), under standard amplification conditions. Relative expression was calculated using the delta-delta Ct method (2^−ΔΔCt^).

### 2.4. Bioinformatic Analysis

Potential ACS-associated miRNAs were identified using miRbase (https://www.mirbase.org/) (accessed on 26 October 2022), miRNet (https://www.mirnet.ca/) (accessed on 26 October 2022), the Human microRNA Disease Database (HMDD, http://www.cuilab.cn/hmdd#fragment–1) (accessed on 26 October 2022) and miRandola (http://mirandola.iit.cnr.it/index.php) (accessed on 26 October 2022). miRNAs present in at least three databases with validated roles in cardiovascular diseases were selected. The analysis focused on identifying miRNAs associated with ACS in the bloodstream, particularly those with previously reported controversial level patterns. This approach aimed to investigate whether these miRNAs could indicate potential transport mechanisms via MVs. The selection included miRNAs that had functional evidence and were validated in the literature for their roles in cardiovascular disease.

### 2.5. Statistical Analysis

All statistical analyses were conducted using appropriate methods based on data distribution. Continuous variables were assessed for normality using the Shapiro–Wilk test: normally distributed variables were reported as mean ± standard deviation (SD) and compared using Student’s *t*-test. Non-normally distributed variables were presented as median and interquartile range (IQR) and compared using the Kruskal–Wallis and Mann–Whitney U tests.

Categorical variables were expressed as absolute frequencies and percentages and analyzed using the Chi-square test or Fisher’s exact test, as appropriate. For miR–126–5p and miR–223–3p, an odds ratio (OR) analysis was performed to evaluate their association with TIMI risk categories. Tertiles were generated for each miRNA, and comparisons were made between the first tertile (T1, lowest levels) and the third tertile (T3, highest levels). The odds ratio (OR) and 95% confidence intervals (CI) were calculated using a logistic regression model and confirmed with Fisher’s exact test. To prevent infinite OR values, a continuity correction was applied when necessary. A *p*-value < 0.05 was considered statistically significant. Analyses were conducted using SPSS v23.0 Software (SPSS Inc., Chicago, IL, USA) and GraphPad Prism Software 8.0.1 (GraphPad Software, La Jolla, CA, USA).

## 3. Results

### 3.1. MVs miRNAs Associated with ACS

**Figure 1:**
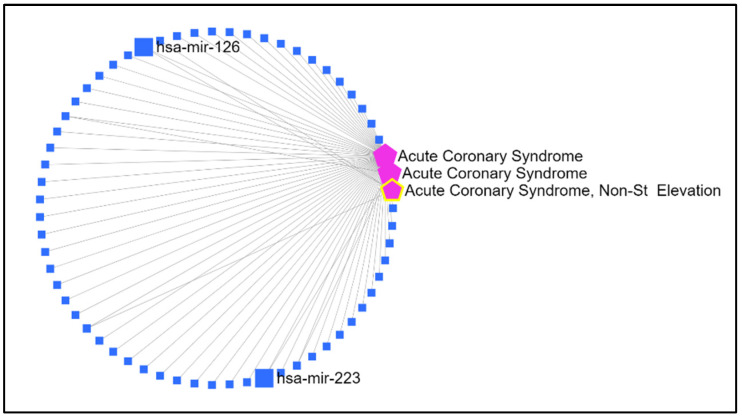
A schematic representation of the network of miRNAs associated with ACS developed using the miRNet database (https://www.mirnet.ca/miRNet/home.xhtml) (accessed on 26 October 2022). The pink pentagons represent the disease (ACS); the squares represent the miRNAs of interest; the lines represent interactions between miRNAs and the disease.

Considering the association with ACS, the analysis revealed that the MV contents of hsa-miR–126–5p and hsa-miR–223–3p were identified in four of the evaluated databases. Figure 1 is a schematic representation of the network obtained from the miRNet database, which highlights the miRNAs selected for this study.

### 3.2. Sociodemographic Data and Clinical Variables of Study Population

The clinical characteristics of the study population were obtained from clinical records and are presented in Table 1.

### 3.3. miR–126–5p and miR–223–3p Levels by TIMI Score

**Figure 2:**
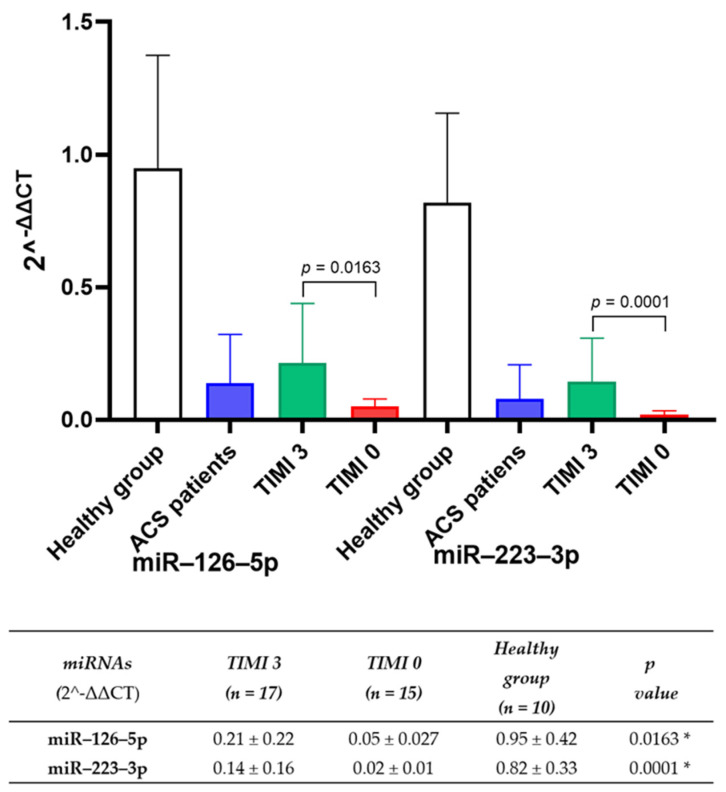
Levels of miR–126–5p and miR–223–3p in the study population. Data represent the mean ± SD. Differences in each miRNA between groups were compared by Student’s *t*-test using the SPSS v23.0 program. A *p*-value < 0.05 was considered statistically significant. * TIMI 3 vs. TIMI 0. miRNA = Small noncoding RNAs; 2^−ΔΔCT^ = delta-delta Ct method.

The 2^−ΔΔCT^ (Figure 2) of each analyzed sample represents the relative level of each of the miRNAs evaluated. miR–126–5p and miR–223–3p levels were significantly lower in TIMI 0 and TIMI 3 patients compared with healthy controls. In TIMI 0 patients, miRNA–126–5p and miRNA–223–3p levels were 4.2 and 7.0 times lower, respectively, compared with TIMI 3 patients.

### 3.4. miR–126–5p and miR–223–3p Levels by Diagnosis

The analysis of miRNA levels by diagnosis showed similar miR–126–5p levels between patients with UA and STEMI, but levels in patients with NSTEMI were 0.9 and 1.2 times higher than in patients diagnosed with UA and STEMI, respectively. In addition, the level of mir–223–3p was similar in all patient groups (Table 2).

### 3.5. Association Analysis of miRNA Levels and TIMI Classification

Analysis of the association of miRNA–126–5p and miRNA–223–3p levels and TIMI classification showed a risk association with both miRNAs (Table 3).

### 3.6. Diagnostic Value of miR–126–5p and miR–223–3p Levels

**Figure 3:**
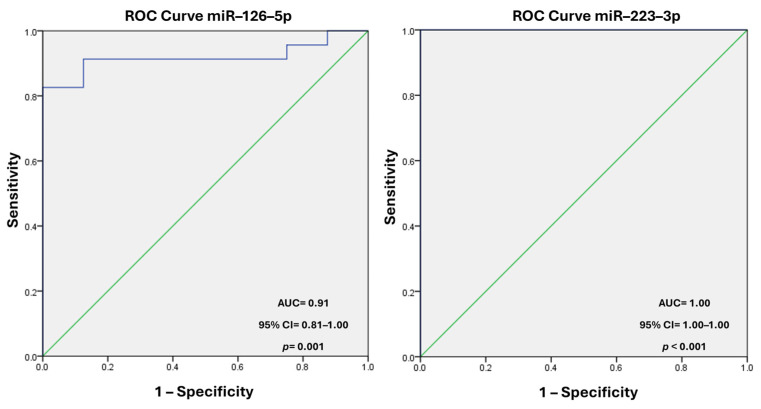
ROC curve of miR–126–5p and miR–223–3p levels in the study population. SPSS v23.0 program. A *p*-value < 0.05 was considered statistically significant. AUC, area under the curve; CI, confidence interval; ROC, receiver operator characteristic.

ROC analysis demonstrated high diagnostic potential, with AUC values of 0.918 for miR–126–5p 95% CI = 0.818–1.00, *p* = 0.001, while for miR–223–3p, the AUC was 1.00 (95% CI = 1.00–1.00, *p* < 0.001), indicating that miR–223–3p has an excellent ability to discriminate between TIMI 0 and TIMI 3 patients (Figure 3).

## 4. Discussion

The development of novel diagnostic strategies for ACS remains at the forefront of cardiovascular research. In our detailed investigation, we examined the potential of two miRNAs—miR–126–5p and miR–223–3p—encapsulated within circulating plasma MVs as biomarkers for ACS, building upon and integrating findings from several influential studies in the field [15,34].

Using the TIMI classification scale, we identified correlations between coronary flow rates and miRNA levels, uncovering their potential as novel biomarkers for risk stratification and therapeutic targeting. Notably, reduced levels of miR-126–5p were associated with lower TIMI scores, suggesting a link between decreased miRNA expression and acute coronary thrombosis events in AMI patients.

These findings underscore the utility of MV-associated miRNAs as innovative biomarkers for long-term risk stratification and support their potential in miRNA-based diagnostics and therapeutics. Key advantages of studying miRNAs within MVs include:

1. Enhanced stability due to RNase protection;
2. Improved specificity, reflecting the physiological state of their cell of origin;
3. Reduced background noise for sharper analytical outcomes; and
4. Superior utility as biomarkers for disease detection and monitoring.

By leveraging these properties, MV-associated miRNAs offer more stable, biologically relevant insights, especially when employed as diagnostic tools or therapeutic targets. 

Recent reviews highlight the significance of MVs in various biological processes [35], reinforcing the importance of analyzing miRNAs within these vesicles.

Bioinformatics tools offer a powerful methodology within omics sciences, facilitating the interpretation of clinical and molecular data [36,37,38]. By integrating bioinformatic analyses with molecular studies, we identified miRNAs consistently associated with ACS, providing robust evidence of their biomarker potential.

In the literature, only a few studies have explored the levels of these miRNAs in ACS, with contradictory findings.

Previous work by Yuan et al. [14] focused on the coronary blood levels of extracellular vesicle-associated miR-126 in patients with AMI compared to those with stable coronary artery disease. They observed that miR-126 levels were significantly lower in AMI patients, and these reduced levels were negatively correlated with TIMI scores, suggesting that diminished miR-126 may be directly linked to acute coronary thrombosis events. Similarly, Massodi et al. [39] investigated several platelet-derived miRNAs—including miR–223–5p, miR–126–5p, miR–484, and miR–130a–3p—in patients with coronary artery disease. Their study proposed that the expression levels of these miRNAs could serve as prognostic biomarkers, especially for ACS subtypes like UA and NSTEMI. Gager et al. [40] contributed to this line of research by reporting a modest increase in plasma miR-223 levels in ACS patients, with distinct differences observed between non-ST-segment elevation ACS and STEMI patients. Ling et al. [41] further enriched the literature by showing that exosomal and serum miR-126 levels were significantly elevated during the acute phase of AMI and UA compared to controls, which underscores the dynamic nature of miRNA expression during cardiovascular events. Additionally, Becker et al. [42] suggested that miR–126 could serve as an effective marker for changes in platelet reactivity in non-ST-elevation ACS patients, while Stojkovic et al. [43] found an association between platelet miR-126 and monocyte–platelet aggregates in ACS patients undergoing antiplatelet therapy. Szelenberger et al. [44] also reported significantly elevated levels of miR–223–3p in blood platelets of ACS patients relative to control subjects, although differences in miR–126–3p were less pronounced. For more details, please refer to the table in the Supplementary Materials.

Even though our results are limited by the lack of a diverse population, focusing on a well-defined group allowed us to explore specific biological patterns following a well-controlled preanalytical phase. This approach minimizes variability stemming from differences in healthcare practices, environmental factors, or sample collection protocols, which can significantly impact miRNA levels and extracellular vesicle signatures [45,46].

Our study extends these findings by focusing on MV-associated miRNAs rather than those derived from plasma or serum [47]. This distinction is critical, as MV-associated miRNAs benefit from enhanced stability; they are protected from degradation by RNases, which ensures that their bioactivity is preserved for reliable diagnostic use. The encapsulation of these miRNAs not only provides protection but also facilitates their targeted delivery to recipient cells [48], where they modulate gene expression and influence key physiological processes such as endothelial integrity, angiogenesis, and platelet activation [47].

Utilizing the TIMI classification scale, we correlated coronary flow rates with the levels of MV-associated miR–126–5p and miR–223–3p. Our findings indicate that patients with lower TIMI scores indicative of more severe coronary flow impairment exhibit significantly reduced levels of these miRNAs. This relationship reinforces the concept that the downregulation of miR–126–5p and miR–223–3p contributes to a pro-thrombotic and dysfunctional endothelial environment characteristic of acute coronary events.

## 5. Conclusions

By integrating the detailed insights from the afore mentioned studies with our novel approach, our work highlights the enhanced diagnostic and prognostic potential of MV-associated miRNAs. Their encapsulation within MVs provides not only improved stability but also a more specific reflection of the pathological processes in ACS. This, in turn, may allow these biomarkers to complement existing risk models and guide therapeutic decisions more effectively.

In conclusion, our study underscores that MV-associated miR–126–3p and miR–223–3p are promising biomarkers for ACS. The integration of our findings with those of previous studies offers a comprehensive understanding of the role these miRNAs play in reflecting coronary flow impairment and disease severity. Future longitudinal studies involving larger, more diverse cohorts are essential to validate these findings further and elucidate the mechanistic roles of these miRNAs, paving the way for their application in precision medicine for the effective management of ACS.

## Figures and Tables

**Table 1:** Sociodemographic data and clinical variables of the study population.

Clinical Variable	Healthy Group (n = 10)	ACS Patients (n = 32)	TIMI 3 (n = 17)	TIMI 0 (n = 15)
Age [years]	60.5 [53.8–64.0]	62.0 [57.0–65.0]	62.0 [57.0–66.0]	62.5 [55.0–65.7]
Male n (%)	7 (70.0)	18 (56.3)	9 (52.9)	9 (60.0)
BMI [kg/m^2^]	26.8 [23.7–29.4]	25.7 [23.4–30.4]	24.9 [22.2–30.1]	25.8 [23.7–31.0]
Laboratory data				
Glucose [mg/dL]	93.5 [83.3–105.3]	117.0 [90.0–168.6]	121.0 [89.0–180.5]	116.0 [92.0–141.9]
Triglyceride [mg/dL]	---	155.0 [104.2–196.8]	125.0 [96.1–183.1]	162.2 [130.0–257.2]
Total cholesterol [mg/dL]	---	150.6 ± 45.2	150.5 ± 47.3	150.6 ± 44.8
LDL [mg/dL]	---	86.9 ± 39.6	84.3 ± 44.9	90.5 ± 32.5
HDL [mg/dL]	---	38.2 ± 11.6	41.0 ± 12.5	34.1 ± 9.5
Platelets [×103/µL]	225.0 [200.0–282.5]	205.0 [163.0–231.0]	205.0 [162.0–258.5]	184.5 [159.2–241.7]
MPV [fL]	8.0 ± 2.1	9.3 ± 1.3	9.2 ± 1.3	9.4 ± 1.4
Hemoglobin [g/dL]	15.7 [14.6–18.0]	15.0 [13.8–16.4]	14.9 [13.3–17.0]	15.0 [13.9–16.4]
Hematocrit [%]	49.0 ± 3.8	43.4 ± 7.7	41.9 ± 8.6	45.2 ± 6.3
McV [fL]	89.7 ± 3.2	91.4 ± 5.8	91.5 ± 7.1	91.3 ± 3.9
McH [pg]	30.2 ± 2.3	32.2 ± 1.8	32.1 ± 1.3	30.2 ± 1.7
CPK-MB [U/mL]	---	4.0 [3.1–82.3]	5.9 [3.2–54.1]	3.3 [2.7–227.2]
Troponin [ng/mL]	---	1.2 [0.4–26.7]	1.1 [0.3–37.4]	5.1 [0.6–16.3]
C-reactive protein [mg/dL]	---	6.2 [1.4–33.7]	8.3 [1.4–63.9]	5.5 [1.6–15.9]
Diabetes n (%)	0 (0.0)	8 (25.0)	6 (35.3)	2 (13.3)

Data are represented by mean ± SD or median (interquartile range), or n and (%) according to data type and distribution (Shapiro–Wilk test). Mann–Whitney U test or Chi-square test with the SPSS v23.0 program. *p* < 0.05 was considered statistically significant. ACS, acute coronary syndrome; BMI, body mass index; LDL, low-density lipoprotein; HDL, high-density lipoprotein; MPV, mean platelet volume; McV, mean corpuscular volume; McH, mean corpuscular hemoglobin; CPK-MB, creatine phosphokinase-mb; TIMI, Thrombolysis in Myocardial Infarction; %, percentage; µL, microliter; fL, phentoliter; g/dL, gram/deciliter; kg/m^2^, kilogram/square metre; mg/dL, milli-gram/deciliter; ng/mL, nano-gram/milliliter; pg, pico-gram; U/mL, units/milliliter.

**Table 2:** miRNA levels in the studied groups.

miRNAs (2^−ΔΔCT^)	UA (*n* = 9)	STEMI (*n* = 9)	NSTEMI (*n* = 14)
miR–126–5p	0.035 [0.024–0.093]	0.042 [0.032–0.075]	0.078 [0.047–0.466]
miR–223–3p	0.017 [0.024–0.093]	0.019 [0.009–0.060]	0.022 [0.013–0.209]

Data are represented as median (interquartile range) according to distribution (Shapiro–Wilk test). Kruskal–Wallis and Mann–Whitney U test with the SPSS v23.0 program. *p* < 0.05 was considered statistically significant. UA = Unstable angina, STEMI = Acute ST-segment elevation myocardial infarction, NSTEMI = Acute non-ST-segment elevation myocardial infarction.

**Table 3:** Logistic regression for miR–126–5p and miR–223–3p levels in TIMI 0 classification.

miRNAs	OR	95% CI	*p* Value
miR–126–5p	7.87	(1.10–56.12)	0.040
miR–223–3p	12.00	(1.58–91.08)	0.022

Comparisons were made between the first tertile (T1, lowest levels) and the third tertile (T3, highest levels). OR = Odds ratio; CI = Confidence intervals.

## Data Availability

The data underlying this article will be shared on reasonable request to the corresponding author.

## Supplementary Materials

The following supporting information can be downloaded at: https://www.mdpi.com/article/10.3390/biomedicines13020510/s1, Table S1: Studies that associate miRNA levels with TIMI, MI, cardiovascular events, and mortality.
