# Supplementary material for: Circulating Microvesicles Enriched in miR–126–5p and miR–223–3p: Potential Biomarkers in Acute Coronary Syndrome
Source: Biomedicines. 2025 Feb 18;13(2):510. doi: 10.3390/biomedicines13020510 (PMC11852751; doi:10.3390/biomedicines13020510)
Supplement: Supplementary file 1 [file biomedicines-13-00510-s001.zip › biomedicines-3453563-supplementary.pdf]

Circulating Microvesicles Enriched in miR–126–5p and miR–223–3p: Potential Biomarkers in Acute Coronary Syndrome

José Rubicel Hernández-López [1], Mirthala Flores-García [2], Esbeidy García-Flores [2,3], Benny Giovanni Cazarín-Santos [1,2], Marco Antonio Peña-Duque [4], Fausto Sánchez-Muñoz [5,6], Martha Alicia Ballinas-Verdugo [6], Hilda Delgadillo-Rodríguez [7], Marco Antonio Martínez-Ríos [8,†], Eduardo Angles-Cano [9,*] and Aurora de la Peña-Díaz [1,2,*]

[1] Pharmacology Department, Faculty of Medicine, National Autonomous University of Mexico,
Circuito Escolar, Ciudad Universitaria, Coyoacán, Mexico City 04510, Mexico; rubicel1981@hotmail.com (J.R.H.-L.); benny.cazarin@facmed.unam.mx (B.G.C.-S.)
[2] Molecular Biology Department, National Institute of Cardiology Ignacio Chávez. Juan Badiano 1, Tlalpan, Mexico City 14080, Mexico; mirthala.flores@cardiologia.org.mx (M.F.-G.); esbeidy.garcia@quimica.unam.mx (E.G.-F.)
[3] Biochemistry Department, Faculty of Chemistry, National Autonomous University of Mexico,
Circuito Escolar, Coyoacán, Mexico City 04510, Mexico
[4] Cardiology Service, Medica Sur. Puente de Piedra 150, Toriello Guerra, Tlalpan, Mexico City 14050, Mexico; marcopduque@gmail.com
[5] Physiology Department, National Institute of Cardiology Ignacio Chávez. Juan Badiano 1, Tlalpan,
Mexico City 14080, Mexico; fausto22@yahoo.com
[6] Immunology Department, National Institute of Cardiology Ignacio Chávez. Juan Badiano 1, Tlalpan,
Mexico City 14080, Mexico; ballinasv75@gmail.com
[7] Department of Hospitalization, National Institute of Cardiology Ignacio Chávez. Juan Badiano 1, Tlalpan, Mexico City 14080, Mexico; hilda_car@yahoo.com
[8] Independent Researcher, Tlalpan, Mexico City 14050, Mexico; mtzrios@gmail.com
[9] INSERM UMR_S-1140 & UMR_S-1144, Innovation Diagnostique et Thérapeutique en Pathologies
Cérébrovasculaires et Thrombotiques, Faculté de Pharmacie de Paris, Université Paris Cité, 75006 Paris, France
* Correspondence: aurorad@unam.mx (A.P.D.); angles-cano@inserm.fr (E.A.C.)
† Retired.

SUPPLEMENTARY MATERIAL

Supplementary Table S1. Studies that associate miRNA levels with TIMI, MI, cardiovascular events, and mortality.

Reference	Study characteristic	miRNA screening	miRNAs studied	Results
Zampetaki et al (2012). [13]	A prospective, population-based survey, included 47 patients diagnosed with MI.	Platelet-derived MVs, platelets and PBMC.	miR-126-3p, miR-197, miR-223.	Significant associations of miR-126 (increasing risk), miR-223 and miR-197 (protective risk) and MI.
Yuan et al (2014). [14]	Included 20 patients with AMI and 20 with SCAD.	Coronary blood extracellular MVs sample.	miR-126	Lower miR-126 levels of EV significant associated in AMI patients in comparison with SCAD). AMI patients, miR-126 levels were negatively associated with TIMI scores.
Jansen et al (2014). [15]	Included 181 patients with SCAD.	Circulating MVs and plasma of arterial blood.	miR-126,miR-222, miR-let7d,miR-21, miR-20a, miR-27a,	Increased expression of miR-126 and miR-199a in circulating MVs with a lower major adverse CV event rate. The other miRNAs, did

			miR-92a, miR-17, miR-130, miR-199a.	not show association with CV events.
Bye et al (2016). [16]	A prospective study, analyzed as case-control with 10-year follow-up; included 212 participants (106 healthy people and 106 patients with fatal AMI), 112 in the derivation cohort and 100 in a separate validation cohort.	Venous blood serum sample.	let-7d-5p,let-7g-5p, miR-26a-5p,miR-29c-3p, miR-101-3p, miR-103a-3p,miR-106a-5p, miR-144-3p, miR-148b-3p,miR-151a-5p, miR-424-5p, miR-660-5p	The combination of miRNAs: 106a-5p, 424-5p, let-7g-5p, 144-3p and 660-5p is the best model to predicting future AMI (with a 78% overall correct classification).
Gigante et al (2020). [17]	A cohort study, included 200 participants; 100 MACE and 100 MACE-free.	Circulating microRNA.	55 miRNAs.	microRNA: 145-3p, 362-3p, 454, 20b, 196b, 185, 301b, 19b1 and 134 associated with an increased risk of MACE and microRNA-720 with a reduced MACE risk.
Keller et al (2017). [18]	Two prospective cohorts, included 178 participants: 21 with CA event and 157 controls.	Peripheral blood.	miR-34a, miR-223, miR-378, miR-499, miR-133.	All-cause 5-year mortality was associated with reduced miR-133 and a trend with lower miR-223 levels. The five miRNAs as panel showed an association with overall mortality.
Velle-Forbord et al. (2019) [19]	Case-control study with a 10-year observation period, 96 cases with MI and 99 controls (healthy participants).	Venous blood serum sample.	let-7g-5p,miR-21-5p, miR-26a-5p,miR-29c-3p, miR-106a-5p, miR-144-3p,miR-151a-5p, miR-191-5p, miR-424-5p and miR-451a.	The combination of miRNAs: 21-5p, 26a-5p, 29c-3p, 144-3p and 151a-5p to the Framingham Risk Score is the best model for predicting 10-year risk of MI.

AMI = Acute myocardial infarction; CAD = Stable coronary artery disease; CV = Cardiovascular; MACE = Major coronary event; MI = Myocardial infarction; MVs = Microvesicles; PBMC= Peripheral blood mononuclear cells.

References:

13. Zampetaki, A.; Willeit, P.; Tilling, L.; Drozdov, I.; Prokopi, M.; Renard, J.M.; Mayr. A.; Weger, S.; Schett, G.; Shah, A.; et al. Prospective study on circulating MicroRNAs and risk of myocardial infarction. J Am Coll Cardiol 2012; 60: 290-299.

14. Yuan, Y.; Ma, Y.; Aili, Z., Nijiati, M. Reductions in extracellular vesicle-associated microRNA-126 levels in coronary blood after acute myocardial infarction: A retrospective study. Front Cardiovasc Med 2022; 9: 1046839.

15. Jansen, F.; Yang, X.; Proebsting, S.; Hoelscher, M.; Przybilla, D.; Baumann, K.; Schmitz, T.; Dolf, A., Endl, E.; Franklin, B.S.; et al. MicroRNA expression in circulating microvesicles predicts cardiovascular events in patients with coronary artery disease. J Am Heart Assoc 2014; 3: e001249.

16. Bye, A.; Røsjø, H.; Nauman, J.; Silva, G.J.; Follestad, T.; Omland, T.; Wisløff, U. Circulating microRNAs predict future fatal myocardial infarction in healthy individuals - The HUNT study. J Mol Cell Cardiol 2016; 97: 162-8.

17. Gigante, B.; Papa, L.; Bye, A.; Kunderfranco, P.; Viviani, C.; Roncarati, R.; Briguori, C.; de Faire, U.; Bottai, M.; Condorelli, G. MicroRNA signatures predict early major coronary events in middle-aged men and women. Cell Death Dis 2020; 11: 74.

18. Keller, T.; Boeckel, J.N.; Groß, S.; Klotsche, J.; Palapies, L.; Leistner, D.; Pieper, L.; Stalla, G.K.; Lehnert, H.; Silber, S.; et al. Improved risk stratification in prevention by use of a panel of selected circulating microRNAs. Sci Rep 2017; 7: 4511.

19. Velle-Forbord, T.; Eidlaug, M.; Debik, J.; Sæther, J.C.; Follestad, T.; Nauman, J.; Gigante, B.; Røsjø, H.; Omland, T.; Langaas, M.; et al. Circulating microRNAs as predictive biomarkers of myocardial infarction: Evidence from the HUNT study. Atherosclerosis 2019; 289: 1-7.
